# Surveillance and Genomic Analysis of Third-Generation Cephalosporin-Resistant and Carbapenem-Resistant *Klebsiella pneumoniae* Complex in Germany

**DOI:** 10.3390/antibiotics11101286

**Published:** 2022-09-21

**Authors:** Kyriaki Xanthopoulou, Can Imirzalioglu, Sarah V. Walker, Michael Behnke, Ariane G. Dinkelacker, Simone Eisenbeis, Petra Gastmeier, Hanna Gölz, Nadja Käding, Winfried V. Kern, Axel Kola, Evelyn Kramme, Kai Lucassen, Alexander Mischnik, Silke Peter, Anna M. Rohde, Jan Rupp, Evelina Tacconelli, David Tobys, Maria J. G. T. Vehreschild, Julia Wille, Harald Seifert, Paul G. Higgins, on behalf of the DZIF R-Net Study Group

**Affiliations:** 1 German Centre for Infection Research (DZIF), 38124 Braunschweig, Germany; 2 Institute for Medical Microbiology, Immunology and Hygiene, Faculty of Medicine and University Hospital Cologne, University of Cologne, 50935 Cologne, Germany; 3 Institute of Medical Microbiology, Justus Liebig University Giessen, 35392 Giessen, Germany; 4 Institute of Hygiene and Environmental Medicine, Charité–Universitätsmedizin Berlin, Corporate Member of Freie Universität Berlin, Humboldt-Universität zu Berlin and Berlin Institute of Health, 12203 Berlin, Germany; 5 National Reference Centre for the Surveillance of Nosocomial Infections, 12203 Berlin, Germany; 6 Institute of Medical Microbiology and Hygiene, University Hospital Tübingen, 72074 Tübingen, Germany; 7 Division of Infectious Diseases, Department of Internal Medicine I, University Hospital Tübingen, 72076 Tübingen, Germany; 8 Institute for Medical Microbiology and Hygiene, University Medical Centre Freiburg, 79104 Freiburg, Germany; 9 Department of Infectious Diseases and Microbiology, University of Lübeck and University Hospital Schleswig-Holstein, Campus Lübeck, 23538 Lübeck, Germany; 10 Division of Infectious Diseases, Department of Medicine II, Faculty of Medicine, Medical Centre, University of Freiburg, 79106 Freiburg, Germany; 11 Department I of Internal Medicine, Faculty of Medicine and University Hospital of Cologne, University of Cologne, 50937 Cologne, Germany; 12 Department of Internal Medicine, Infectious Diseases, University Hospital Frankfurt, Goethe University Frankfurt, 60590 Frankfurt am Main, Germany

**Keywords:** *Klebsiella pneumoniae* complex, colonisation, bloodstream infections, third-generation cephalosporin resistance, carbapenem resistance, typing

## Abstract

To analyse the epidemiology and population structure of third-generation cephalosporin-resistant (3GCR) and carbapenem-resistant (CR) *Klebsiella pneumoniae* complex isolates, patients were screened for rectal colonisation with 3GCR/CR *K. pneumoniae* complex on admission to six German university hospitals (2016–2019). Also collected were 3GCR/CR and susceptible *K. pneumoniae* isolates from patients with bloodstream infections (2016–2018). Whole-genome sequencing was performed followed by multilocus sequencing typing (MLST), core-genome MLST, and resistome and virulome analysis. The admission prevalence of 3GCR *K. pneumoniae* complex isolates during the 4-year study period was 0.8%, and 1.0 bloodstream infection per 1000 patient admissions was caused by *K. pneumoniae* complex (3GCR prevalence, 15.1%). A total of seven *K. pneumoniae* complex bloodstream isolates were CR (0.8%). The majority of colonising and bloodstream 3GCR isolates were identified as *K. pneumoniae*, 96.7% and 98.8%, respectively; the remainder were *K. variicola* and *K. quasipneumoniae*. cgMLST showed a polyclonal population of colonising and bloodstream isolates, which was also reflected by MLST and virulome analysis. CTX-M-15 was the most prevalent extended-spectrum beta-lactamase, and 29.7% of the colonising and 48.8% of the bloodstream isolates were high-risk clones. The present study provides an insight into the polyclonal 3GCR *K. pneumoniae* population in German hospitals.

## 1. Introduction

The Gram-negative bacterium *Klebsiella pneumoniae*, a member of the family Enterobacterales, is a natural inhabitant of the gastrointestinal tract of humans and animals. It is also encountered as a major cause of hospital- and community-acquired infections, such as urinary and respiratory tract infections, as well as bloodstream infections (BSI) [1,2]. The World Health Organisation listed third-generation cephalosporin-resistant (3GCR) and carbapenem-resistant (CR) *K. pneumoniae* in 2019 as a “Priority 1: Critical group” organism for which new antimicrobials are urgently needed [3]. Recently, the taxonomic classification of the *K. pneumoniae* complex, including seven phylogroups, has been revised. On the basis of population studies, the *K. pneumoniae* complex has been expanded, encompassing the bacterial species *K. pneumoniae*, *K. quasipneumoniae* subsp. *quasipneumoniae*, *K. quasipneumoniae* subsp. *similipneumoniae*, *K. variicola* subsp. *variicola*, *K. variicola* subsp. *tropica*, *K. quasivariicola*, and *K. africana* [4,5,6,7,8].

The rapid expansion of 3GCR and CR *K. pneumoniae* is an increasing public health threat in Europe. In 2020, 3GCR rates of invasive *K. pneumoniae* isolates were 50% or above in 20% of the countries reporting data to the European Antimicrobial Resistance Surveillance network (EARS-Net), mainly in Southern and Eastern Europe [9]. Furthermore, an admission prevalence study in Germany in 2014 reported a carriage rate of 9.5% for 3GCR Enterobacterales and 0.8% for *K. pneumoniae* [10]. The 3GCR phenotype is mainly attributed to the presence of extended-spectrum beta-lactamases (ESBLs), such as *bla*_CTX-M-15_, AmpC beta-lactamases, SHV hyperproduction, and decreased outer membrane permeability [11,12,13]. In addition, carbapenem resistance in *K. pneumoniae* mainly mediated by KPC, NDM, OXA-48, and VIM remains at relatively low levels for most European countries including Germany (0.5% in 2020) [9,14]. However, a North/South gradient is evident concerning CR *K. pneumoniae*, with higher resistance rates observed in Mediterranean countries (e.g., Greece and Italy) compared to Northern European countries [9]. *K. pneumoniae* is the major species within the complex; nevertheless, other members such as *K. variicola* or *K. quasipneumoniae* subsp. *quasipneumoniae* may also be involved in human infections and associated with carbapenemases (such as *bla*_NDM-9_ and *bla*_OXA-181_) and are gaining more recognition [15,16,17,18]. 

The burden of antimicrobial resistance in the hospital environment is tightly linked to the successful spread of certain bacterial clones, known as high-risk (HiR) clones, that are isolated in different geographic locations at different times and are more likely to cause outbreaks [19,20]. Numerous multidrug-resistant (MDR) or extensively drug-resistant (XDR) *K. pneumoniae* HiR clones have been identified as causing outbreaks around the world [1,21]. The presence of certain plasmids has contributed to the success of some HiR clones, e.g., sequence type (ST) 258 has spread worldwide after the acquisition of a *bla*_KPC_-encoding plasmid [1,22]. Furthermore, HiR clones have been reported harbouring hybrid plasmids containing both resistance and virulence genes [23]. Virulence determinants may enable *K. pneumoniae* to overcome physical and chemical barriers and evade host defence. Cases of hypervirulent strains, which are more virulent than classical *K. pneumoniae* strains causing severe infections in healthy individuals from the community, have been previously reported [24,25,26]. The capsule of *K. pneumoniae* is a well-known virulence factor, since it facilitates colonisation and infection by providing defence against the complement system and macrophages [27,28]. In addition, *K. pneumoniae* may gain an advantage over host cells in acquiring iron by expressing iron scavenging systems such as aerobactin and enterobactin [29,30].

A surveillance study was conducted as part of the multicentre Resistance-Network study (R-Net) of the German Centre for Infection Research (DZIF) at six German university hospitals. To understand the German-wide epidemiology and population structure of 3GCR and CR *K. pneumoniae* complex isolates, we conducted a genome-based analysis that included isolates from patients colonised on hospital admission, as well as invasive isolates from hospitalised patients with BSI, and focused on the molecular characterisation and the identification of HiR clones and resistance and virulence genes. Because of the large scale of the present multicentre study, the data gained about the colonising and bloodstream *K. pneumoniae* complex isolates will fill the knowledge gap about the genotypes circulating in the country, the molecular epidemiology and will provide data that could strengthen effective infection control measures.

## 2. Results

### 2.1. Antimicrobial Susceptibility and Species Distribution

**Figure 1:**
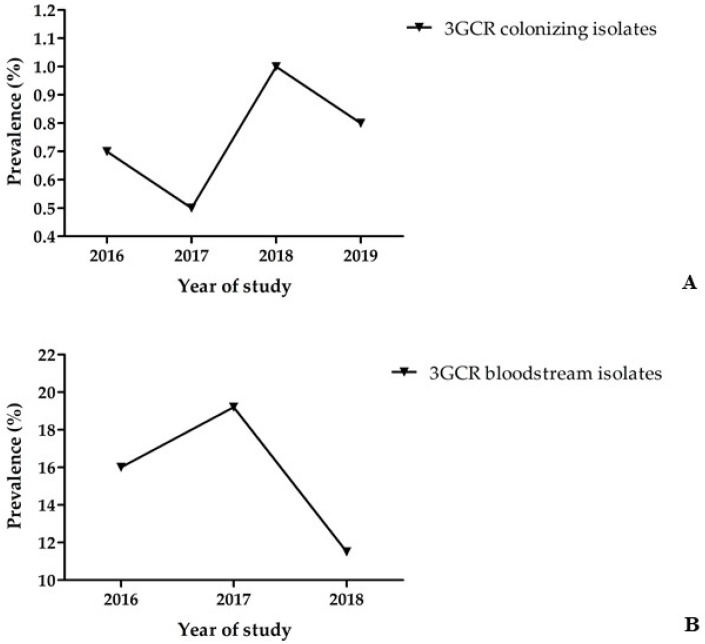
(**A**) Prevalence of 3GCR *K. pneumoniae* complex carriage of patients on hospital admission. (**B**) Prevalence of 3GCR among 880 *K. pneumoniae* complex bloodstream isolates.

Between 2016 and 2019, a total of 11,885 patients were screened for rectal colonisation with 3GCR/CR *K. pneumoniae* complex isolates within 3 days of hospital admission. Of these, 92 patients were found colonised with 3GCR *K. pneumoniae* complex isolates on hospital admission, accounting for a prevalence of 0.8% (Table 1). Overall, no increase was observed in the prevalence of 3GCR/CR *K. pneumoniae* complex isolates on hospital admission between 2016 and 2019 (2016, 0.7%; 2017, 0.5%; 2018, 1%; 2019, 0.8%) at the six study centres (Figure 1A and Supplementary Figure S1A). Among 3GCR isolates, 98.9% (*n* = 91) were resistant to cefotaxime and 69.6% (*n* = 64) to ceftazidime. A total of 91 *K. pneumoniae* complex isolates were available for molecular characterisation using WGS. Genotyping identified 88 colonising isolates as *K. pneumoniae* (96.7%), 2 isolates as *K. quasipneumoniae* subsp. *quasipneumoniae* (2.2%), and 1 isolate as *K. variicola* subsp. *variicola* (1.1%). 

Within the surveillance study of nosocomial BSI, a total of 880 *K. pneumoniae* complex bloodstream isolates (Table 1) were recovered from patients at the six study centres between 2016 and 2018, accounting for an incidence of 1.0 per 1000 patient admissions (0.21 *K. pneumoniae* complex BSI per 1000 patient days). The incidence of 3GCR *K. pneumoniae* complex BSI was 0.15 per 1000 patient admissions (0.03 3GCR *K. pneumoniae* complex BSI per 1000 patient days). A total of 133 *K. pneumoniae* complex isolates were 3GCR, accounting for a prevalence of 15.1% (2016, 16%; 2017, 19.2%; 2018, 11.5%; Figure 1B and Supplementary Figure S1B), while only seven isolates were CR (prevalence, 0.8%). Among the 3GCR BSI isolates, 94% of isolates (*n* = 125) were resistant to cefotaxime, while 90.2% (*n* = 120) were resistant to ceftazidime, and 4.5% (*n* = 6) and 5.3% (*n* = 7) were resistant to imipenem and meropenem, respectively. Of 133 3GCR/CR isolates, 80 were available for molecular characterisation, of which, 79 isolates were identified as *K. pneumoniae* (98.8%) and 1 isolate as *K. variicola* subsp. *variicola* (1.2%). Finally, between 2016 and 2018, a total of 721 bloodstream infections (Table 1) were caused by 3GCS/CS *K. pneumoniae* complex, while another 26 bloodstream infections involved *K. pneumoniae* complex isolates that tested intermediate (cefotaxime MIC, 2 mg/L, and/or ceftazidime MIC, 2–4 mg/L; Table 1). Ninety-five 3GCS/CS isolates were subjected to WGS and molecular typing, and of these, 71 were identified as *K. pneumoniae* (74.7%), 20 as *K. variicola* subsp. *variicola* (21.1%), 3 isolates as *K. quasipneumoniae* subsp. *similipneumoniae* (3.2%), and 1 isolate as *K. quasipneumoniae* subsp. *quasipneumoniae* (1.1%).

### 2.2. MLST, HiR Clones, and cgMLST Analysis

**Figure 2:**
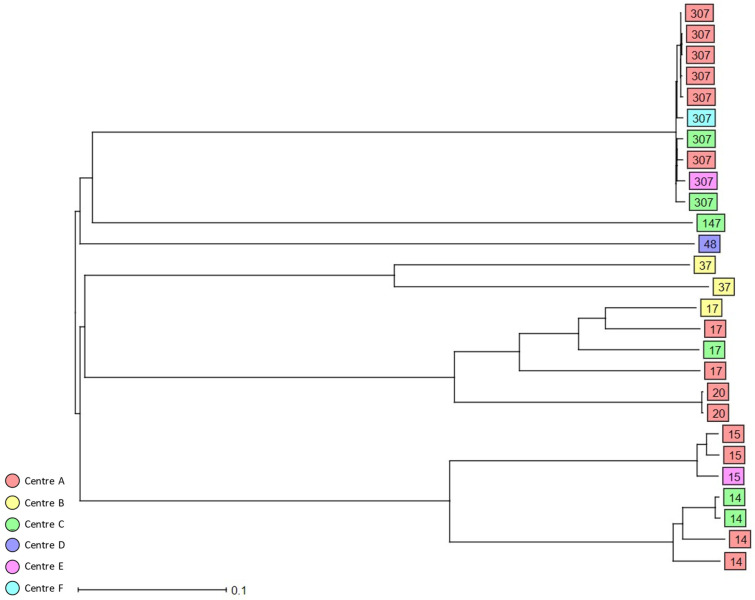
Dendrogram generated using Ridom SeqSphere+ for the HiR 3GCR/CR colonising *K. pneumoniae* complex isolates (*n* = 27) coloured by study centre and identified by ST type, ignoring missing values. Each box represents one isolate from an individual patient according to sequence analysis of 2358 cgMLST target genes.

Using 7-loci MLST, 91 3GCR *K. pneumoniae* complex isolates colonising patients on hospital admission were grouped into 58 STs (Table 2, Supplementary Tables S1 and S2). The most prevalent ST was ST307 (*n* = 10, 11%), followed by ST45 (*n* = 5, 5.5%) and ST219 (*n* = 5, 5.5%). Moreover, 29.7% of the identified STs were classified as HiR clones, i.e., ST307 (*n* = 10), ST14 (*n* = 4), ST17 (*n* = 4), ST15 (*n* = 3), ST20 (*n* = 2), ST37 (*n* = 2), ST48 (*n* = 1), and ST147 (*n* = 1). cgMLST analysis of the colonising isolates revealed only small clusters of closely related isolates including HiR clone ST307 (*n* = 6) recovered from study centres A and F in 2017, 2018, and 2019; a cluster of three ST1653 isolates from study centre A collected in 2019; and a cluster of three ST219 isolates from centres B, C, and D. Finally, two additional HiR clone clusters were detected, ST14 (*n* = 2) in study centre C and ST20 (*n* = 2) in study centre A (Figure 2 and Supplementary Figure S2). No clusters of closely related isolates were observed for the *K. variicola* and *K. quasipneumoniae* colonising isolates.

**Figure 3:**
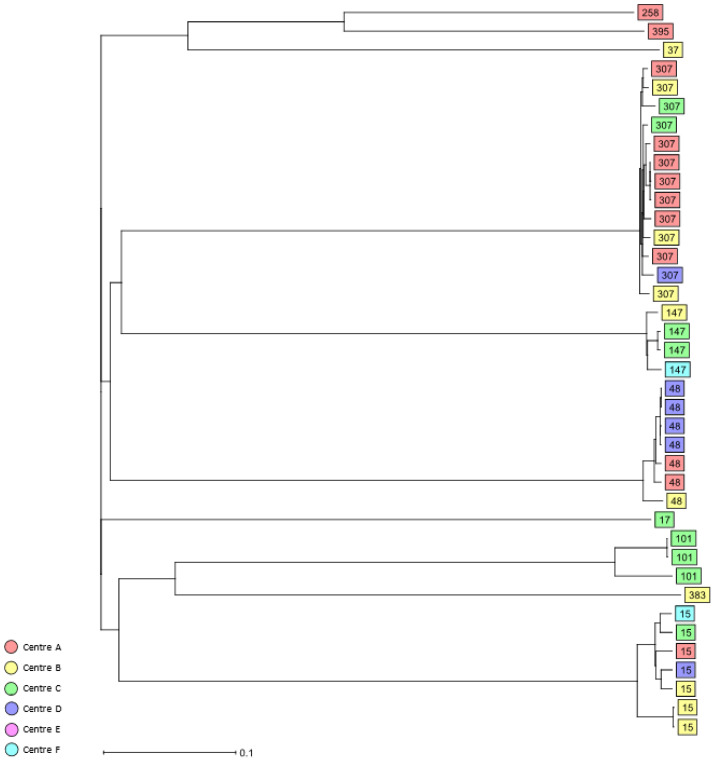
Dendrogram generated using Ridom SeqSphere+ for the HiR 3GCR/CR *K. pneumoniae* complex bloodstream isolates (*n* = 39) coloured by study centre and identified by ST type, ignoring missing values. Each box represents one isolate from an individual patient according to sequence analysis of 2358 cgMLST target genes.

Among the 3GCR/CR *K. pneumoniae* complex bloodstream isolates, 39 different STs were identified (Table 2, Supplementary Tables S1 and S2), the most common being HiR clone ST307 (*n* = 13, 16.2%), HiR clone ST15 (*n* = 7, 8.8%), HiR clone ST48 (*n* = 7, 8.8%), ST219 (*n* = 5, 6.3%), and HiR clone ST147 (*n* = 4, 5%) (Table 2). Other HiR clones identified were ST101 (*n* = 3), ST17 (*n* = 1), ST37 (*n* = 1), ST258 (*n* = 1), ST383 (*n* = 1), and ST395 (*n* = 1). Using cgMLST analysis, only a few clusters of closely related isolates could be identified, including a cluster of five *K. pneumoniae* bloodstream isolates assigned as ST219 that were recovered from three study centres, A, B, and C; a cluster of four HiR clone ST48 isolates recovered in 2017 from study centre D; and a cluster of four HiR clone ST307 isolates recovered from study centre A in 2017 and 2018. Finally, six small clusters of two *K. pneumoniae* isolates each, namely, ST13 (centre A and B), HiR clone ST15 (centre B), HiR clone ST101 (centre C), HiR clone ST147 (centre C), ST607 (centre F), and ST1825 (centres A and C), were also detected in the present study (Figure 3 and Supplementary Figure S3).

**Figure 4:**
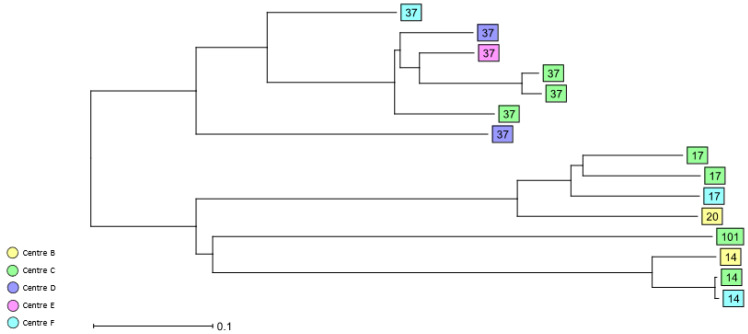
Dendrogram generated using Ridom SeqSphere+ for the HiR 3GCS/CS *K. pneumoniae* complex bloodstream isolates (*n* = 15) coloured by study centre and identified by ST type, ignoring missing values. Each box represents one isolate from an individual patient according to sequence analysis of 2358 cgMLST target genes.

A total of 77 STs were identified among the 3GCS and CS bloodstream isolates (Table 2, Supplementary Tables S1 and S2). HiR clone ST37 (*n* = 7, 7.4%) was the most common ST, followed by HiR clones ST14 and ST17 (each; *n* = 3, 3.2%). The HiR clones ST20 and ST101 were singletons. cgMLST analysis revealed the presence of only a few clusters of closely related 3GCS/CS *K. pneumoniae* isolates. Two ST160 isolates from study centre B and C and recovered in 2017 and 2018, respectively, clustered together, as well as two HiR clone ST14 isolates from centres C and F. Furthermore, two ST3640 isolates recovered from two different patients in centre C in 2018 were identical by cgMLST. Finally, two ST6069 *K. pneumoniae* isolates from centre D recovered in 2017 and 2018 were identical (Figure 4 and Supplementary Figure S4).

### 2.3. Acquired Beta-Lactamases

Four families of beta-lactam resistance determinants were identified among the colonising 3GCR *K. pneumoniae* complex isolates, namely, *bla*_CTX-M-like_ (*n* = 84), *bla*_TEM-like_ (*n* = 47), *bla*_OXA-1_ (*n* = 24), and *bla*_DHA-1_ (*n* = 2) (Table 3). Among ESBL CTX-M beta-lactamases, the most predominant group was *bla*_CTX-M-15_ (*n* = 68), followed by *bla*_CTX-M-14_ (*n* = 11), *bla*_CTX-M-1_ (*n* = 2), *bla*_CTX-M-27_ (*n* = 1), *bla*_CTX-M-55_ (*n* = 1), and *bla*_CTX-M-65_ (*n* = 1). Furthermore, among the broad-spectrum TEM-1 beta-lactamases, *bla*_TEM-1B_ (*n* = 46) was the most common variant identified, and *bla*_TEM-1A_ was detected in one isolate. Finally, one ST1599 *K. variicola* isolate recovered from study centre A was CS and therefore excluded from the study but harboured *bla*_OXA-181_, an OXA-48-like carbapenemase.

A total of seven families of acquired beta-lactamases (Table 3) were detected in the 3GCR *K. pneumoniae* complex bloodstream isolates, namely, *bla*_CTX-M-like_ (*n* = 65), *bla*_TEM-like_ (*n* = 38), *bla*_OXA-like_ (*n* = 36), *bla*_DHA-1_ (5), *bla*_CMY-4_ (*n* = 1), *bla*_KPC-2_ (*n* = 1), and *bla*_VIM-19_ (*n* = 1). *bla*_CTX-M-15_ (*n* = 59) was the predominant variant among the *bla*_CTX-M-like_ ESBLs, followed by *bla*_CTX-M-14_ (*n* = 3), *bla*_CTX-M-1_ (*n* = 1), *bla*_CTX-M-3_ (*n* = 1), and *bla*_CTX-M-27_ (*n* = 1). The ESBL *bla*_OXA-1_ was detected in 30 bloodstream isolates, while four isolates harboured the narrow-spectrum beta-lactamase *bla*_OXA-9_ and two isolates encoded the carbapenemase *bla*_OXA-48_. Furthermore, the broad-spectrum TEM beta-lactamase *bla*_TEM-1B_ (*n* = 35) was the most common *bla*_TEM-like_ variant identified, and *bla*_TEM-1A_ was detected in three isolates. Finally, two families of acquired beta-lactamases were identified among the 3GCS/CS *K. pneumoniae* complex isolates: broad-spectrum beta-lactamase TEM, *bla*_TEM-1A_ (*n* = 1), and *bla*_TEM-1B_ (*n* = 2), and narrow-spectrum beta-lactamase OXA, *bla*_OXA-9_ (*n* = 1).

### 2.4. Virulence Genes 

Since various virulence properties may contribute to infectivity and persistence of *K. pneumoniae*, several virulence-associated factors were analysed in the present study. Forty-nine 3GCR/CR colonising isolates were grouped into 28 different KL types, and 42 isolates were classified as unknowns (Table S2). KL102 (*n* = 9) was the most prevalent K locus and was identified in 6 of 10 HiR clone ST307 and three of three ST1653 colonising isolates. Furthermore, KL2 (*n* = 3) associated with biosynthesis of the K2 capsule serotype was identified in three of four HiR clone ST14. Finally, one of five ST45, one of one HiR clone ST48, and one of one ST791 were typed as KL62 (*n* = 3). The siderophore yersiniabactin *ybt* locus was identified in 41 3GCR/CR *K. pneumoniae* complex isolates. The most prevalent yersiniabactin locus was *ybt* 10 harboured by the integrative and conjugative element ICE*Kp4* (*n* = 10), followed by ICE*Kp3* harbouring *ybt* 9 (*n* = 6), ICE*Kp5* harbouring *ybt* 14 (*n* = 6), and plasmid-encoded *ybt* 4 (*n* = 4). Two colonising isolates encoded the yersiniabactin *ybt* 17 co-located in ICE*Kp10* with colibactin *clb* 3, while another isolate harboured the aerobactin locus *icu 3*. 

The virulence gene content of the 3GCR/CR *K. pneumoniae* bloodstream isolates was also investigated. A total of 20 bloodstream isolates were grouped into 11 KL types, while 60 isolates were classified as unknown (Table S2). KL62 (*n* = 6) was the most common locus associated with the K62 capsular serotype and was identified only in HiR clone ST48 isolates. In addition, KL102 (*n* = 3) was carried by HiR clone ST307, KL24 (*n* = 2) by HiR clone ST15, and KL114 (*n* = 2) by ST219. A total of 34 3GCR/CR isolates carried a siderophore yersiniabactin *ybt* lineage. The most common locus was *ybt* 10 harboured by ICE*Kp4* (*n* = 18), while ICE*Kp3* encoding *ybt* 9 and ICE*Kp12* carrying *ybt* 16 were found in five and three bloodstream isolates, respectively. In addition, an ST25 isolate with unknown KL type carried the aerobactin *icu 1* and salmochelin *iro 1* loci. In the present study, a total of 14 KL loci were identified among the susceptible bloodstream isolates, while 77 were classified as unknown (Table S2). KL2 (*n* = 4) was the most common capsule type identified in two of four HiR clone ST14, one of two ST39, and one of one ST380 isolates followed by KL14 (*n* = 2) in one of seven HiR clone ST37 and one of one ST76 isolates. A total of 32 3GCS/CS *K. pneumoniae* isolates harboured the virulence locus yersiniabactin, and the most common was *ybt* 9 harboured by ICE*Kp3* (*n* = 6), followed by *ybt 10* encoded by ICE*Kp4* (*n* = 5) and *ybt 14* carried by ICE*Kp5* (*n* = 4), while five 3GCS/CS bloodstream isolates harboured a colibactin locus. Furthermore, five *K. pneumoniae* isolates harboured aerobactin loci and six isolates salmochelin, while a total of three isolates harboured the hypermucoidy locus *rmpADC.*

## 3. Discussion

Increasing rates of 3GCR *K. pneumoniae* causing bloodstream infections have been reported by several countries according to EARS-Net. In detail, in 2020, 15% of the reporting countries 3GCR rates were <10%, while in 44% of the countries reporting data to EARS-Net, resistance rates were ≥50%, including mainly countries in the southern and eastern parts of Europe, e.g., Bulgaria (79.1%), Greece (74.5%), and Romania (67.9%) [9]. In the present multicentre surveillance study addressing the prevalence of 3GCR *K. pneumoniae* complex colonising patients on hospital admission, the carriage rate over the four-year study period remained stable, ranging between 0.5 and 1%. This result is in agreement with a previous survey conducted in Germany in 2014 reporting an admission prevalence of 0.8% for 3GCR *K. pneumoniae* [10]. In the present three-year surveillance study of patients with bloodstream infections, the prevalence of 3GCR isolates of *K. pneumoniae* complex was found to be 15.1% (2016, 16%; 2017, 19.2%; 2018, 11.5%), showing a slight decrease over the study period. Resistance to third-generation cephalosporins was mainly mediated by *bla*_CTX-M-like_ betalactamases, with no difference between colonising and bloodstream isolates. EARS-Net reported for Germany slightly lower 3GCR rates in *K. pneumoniae* isolates causing invasive infections, i.e., 13.6% in 2016, 14.6% in 2017, and 12.9% in 2018 [9]. Finally, no colonising CR *K. pneumoniae* complex isolates were detected in the present study, while the prevalence of carbapenem resistance among bloodstream isolates was rather low (0.8%). Similar results were also reported by EARS-Net and other studies, suggesting that carbapenem resistance in *K. pneumoniae* complex is currently not a threat in Germany [9,10]. 

In the present study, the vast majority of 3GCR/CR bloodstream isolates (98.8%) were *K. pneumoniae*, confirming that this species is the major cause of invasive *K. pneumoniae* complex infections in healthcare settings in Germany [4,31]. Similarly, 96.7% of the colonising isolates were identified as *K. pneumoniae*. However, 25.4% 3GCS/CS bloodstream isolates presumptively identified as *K. pneumoniae* by the participating study centres were re-identified as non-*K. pneumoniae* by sequencing, highlighting that susceptible *K. variicola* and *K. quasipneumoniae* isolates are often causing bloodstream infections in Germany and are underreported. Similar frequencies of non-*K. pneumoniae* bloodstream infections, i.e., 24.4% *K. variicola* and 6.5% *K. quasipneumoniae*, were reported in the Stockholm area [16].

Overall, both colonising and bloodstream *K. pneumoniae* complex isolates were classified into a large number of STs (3GCR/CR colonising, *n* = 58; 3GCR/CR, *n* = 39; and 3GCS/CS, *n* = 77 bloodstream isolates), indicating that the population structure of *K. pneumoniae* in German hospitals is heterogenous and consists of multiple genotypes with even more diversity among 3GCS/CS vs. 3GCR/CR bloodstream isolates. *K. pneumoniae* HiR ST307 was the most frequent clone among the 3GCR/CR colonising and bloodstream isolates in the present study, accounting for 10 (11%) and 13 (16.2%) of isolates, respectively. HiR ST307 has a global distribution and has caused numerous outbreaks in healthcare settings worldwide [32]. Furthermore, an increasing prevalence has been observed for ST307 carbapenemase-producing *K. pneumoniae* isolates, even replacing other successful HiR clones such as ST258 in Italy and Colombia [21,33]. Outbreaks of XDR ST307 carrying resistance plasmids encoding either NDM-1, CTX-M-15, or OXA-48, have been reported previously in four medical facilities in north-eastern Germany [34,35]. Among the 3GCR colonising *K. pneumoniae* isolates, 29.7% were identified as HiR, as were 48.8% of the 3GCR/CR bloodstream isolates. When only the ST is used as inclusion criterion and the susceptible phenotype is not taken into account, HiR clones were also identified among 3GCS/CS bloodstream isolates, including ST14, ST17, ST20, ST37, and ST101, accounting for 15.8% of these isolates. These data suggest that although various *K. pneumoniae* HiR clones are circulating in the six German university hospitals participating in the study, no particular clone was predominant. 

The cgMLST analysis of both colonising and bloodstream *K. pneumoniae* complex isolates revealed only a few small clusters of closely related *K. pneumoniae* isolates, indicating potential transmission events, whereas the non-*K. pneumoniae* isolates were all singletons. Representatives of HiR clones such as ST13, ST15, ST48, ST101, ST147, and ST307 also formed small clusters comprising between two and six isolates that were mainly centre-specific and likely reflect the local epidemiology of each study centre, while isolates representing other well-known HiR clones such as ST258 were singletons. Numerous previous studies have reported the presence of HiR clones such as ST101, ST147, or ST258 in different hospitals in Germany, highlighting that these strains were already circulating in the country [36,37,38]. 

Resistance to 3GC was caused mainly by ESBLs of the CTX-M-1 group (CTX-M-1, CTX-M-3, CTX-M-15), found in 76.9% of 3GCR colonising and 76.2% of bloodstream isolates, and of the CTX-M-9 group (CTX-M-14, CTX-M-27, CTX-M-55, CTX-M-65), found in 15.4% of colonising and 5% of bloodstream isolates. Consistent with our results, the CTX-M-1 group was the predominant ESBL group in *K. pneumoniae* in a survey of admission prevalence in Germany in 2014 and 2015 as also the case in other regions of Europe [10,39,40]. The high prevalence of ESBLs, such as CTX-M-15, in the polyclonal population of colonising and bloodstream isolates of the present study may suggest that horizontal plasmid transmission or dissemination of other mobile genetic elements may be responsible for the spread of acquired beta-lactamases, which needs to be further investigated. Of note, three of four carbapenemases detected in bloodstream isolates at study sites B and C were found in HiR clones, i.e., OXA-48, ST101; KPC-2, ST258; and VIM-19, ST383, which also co-harboured ESBLs and other antimicrobial resistance genes. In Enterobacterales, OXA-48, VIM-1, and KPC-2 are the most frequently detected carbapenemases in Germany, as reported by the German National Center for Multidrug-Resistant Gram-Negative Bacteria and have been frequently involved in healthcare-associated infections and outbreaks [37,38,41,42].

Numerous studies have reported that hypervirulent *K. pneumoniae*, a pathotype that is more virulent than classical *K. pneumoniae*, carries large virulence plasmids and causes infections in healthy individuals in the community. Furthermore, increased virulence has been associated with certain capsular serotypes, e.g., K1 and K2 [4,24,26]. In the present study, KL102 was the most frequent capsule locus among the colonising isolates, mainly detected in HiR ST307 (67%), concurring with previous findings from a *K. pneumoniae* ST307 outbreak in north-eastern Germany [35]. Conversely, KL62 was the most frequent capsular serotype among 3GCR/CR bloodstream isolates and was only detected in HiR ST48 isolates as previously reported in China [43]. Different acquired virulence factors such as capsule and aerobactin production have been linked to hypervirulent strains [24,26]. Siderophores such as yersiniabactin were identified in the present study in 45%, 42.5%, and 33.7% of the 3GCR/CR colonising, 3GCR/CR, and 3GCS/CS bloodstream isolates, respectively. Furthermore, aerobactin, colibactin, salmochelin, and the hypermucoidy determinant *rmpA* were also detected in a small number of isolates. In the present study, the diverse population structure of *Klebsiella* spp. isolates was also confirmed by the heterogeneity of acquired virulence factors.

To the best of our knowledge, the present work represents the largest study on the molecular epidemiology of 3GCR/CR colonising and bloodstream *K. pneumoniae* complex isolates in Germany; however, our study has several limitations. The data obtained from the admission prevalence study are not representative of the general population because many patients admitted to tertiary care hospitals represent a patient population with frequent hospital contacts and colonisation may have occurred during previous healthcare contacts. In addition, the present study did not investigate whether 3GCR/CR bloodstream infections were caused by the same isolates found at hospital admission.

In summary, the results of the present study describe the polyclonal population of 3GCR *K. pneumoniae* complex isolates that colonise patients on hospital admission and cause bloodstream infections. Although certain *K. pneumoniae* genotypes were more frequent, no extensive clonality was detected among the isolates, suggesting that a diverse 3GCR *K. pneumoniae* population circulates in German hospitals. MLST and virulome analysis supported these findings. Finally, our data demonstrate the presence of HiR clones circulating in Germany and provide epidemiological data on 3GCR and CR *K. pneumoniae* complex isolates that may contribute to more effective infection control.

## 4. Materials and Methods

### 4.1. Study Participants and Design

Two independent epidemiologic surveys involving patients (i) at hospital admission and (ii) with nosocomial BSI were conducted at six German tertiary care university hospitals in eastern (centre A), western (centre B), south-western (centres C and F), central (centre D), and northern (centre E) Germany.

In the prospective epidemiological study on the prevalence of 3GCR and/or CR (3GCR/CR) *K. pneumoniae* complex on hospital admission, patients aged ≥18 years were included between June 2016 and December 2019. Centre F did not participate in the survey in 2016. Excluded were patients from the departments of ophthalmology, paediatrics, psychiatry, and from intensive care units (ICUs). 3GCR/CR *K. pneumoniae* complex bloodstream isolates were collected from patients aged ≥18 years between October 2016 and December 2018. Patients from the departments of ophthalmology, paediatrics, and psychiatry were excluded. Finally, a random sample of third-generation cephalosporin-susceptible (3GCS) and carbapenem-susceptible (CS) *K. pneumoniae* complex bloodstream isolates collected between 2016 and 2018 at the same study centres were used as controls. 

### 4.2. Species Identification and Antimicrobial Susceptibility Testing

Species identification was performed using MALDI-TOF MS (Bruker Daltonics GmbH, Bremen, Germany). Antimicrobial susceptibility testing (AST) for cefotaxime, ceftazidime, imipenem, and meropenem was carried out using VITEK^®^2 (bioMérieux, Nürtingen, Germany). VITEK^®^2 was routinely validated using characterised quality control strains. Isolates that were non-susceptible to cefotaxime and/or ceftazidime (MIC ≥ 2 mL/L) as well as isolates non-susceptible to imipenem and/or meropenem (MIC ≥ 4 mL/L) were included in the study and further characterised on the basis of the EUCAST resistance breakpoints for Enterobacterales (Version 6.0, January 2016, http://www.eucast.org/, accessed on 1 April 2022).

### 4.3. Whole-Genome Sequencing (WGS)

Total DNA was extracted using the MagAttract HMW DNA Kit (Qiagen, Hilden, Germany) according to the manufacturer’s instructions. Sequencing libraries were prepared using the Nextera XT library prep kit (Illumina GmbH, Munich, Germany) for a 250 bp paired-end sequencing run on a MiSeq (Illumina GmbH) platform. The genomes were assembled *de novo* using Velvet [44]. 

### 4.4. Molecular Species Identification, Molecular Epidemiology, and High-Risk Clones

The species-intrinsic beta-lactamase gene (*bla*_SHV_ in *K. pneumoniae*, *bla*_LEN_ in *K. variicola*, *bla*_OKP_ in *K. quasipneumoniae*) were used as indicators for species identification. Furthermore, the genotyping tool Kleborate (https://github.com/katholt/Kleborate, accessed on 1 March 2022) was used to identify the species using genome assemblies [31]. Finally, JSpeciesWS was used to further confirm the species of the isolates identified as non-K. *pneumoniae* [45]. The *Klebsiella* spp. Multi-locus sequence typing (MLST) scheme was used to assign the ST (https://bigsdb.web.pasteur.fr/, accessed on 1 March 2022). The molecular epidemiology was investigated using a *K. pneumoniae sensu lato* core genome MLST (cgMLST) scheme (https://www.ridom.de/seqsphere/u/Task_Template_Sphere.html, accessed on 1 April 2022), including 2358 target alleles, using the Ridom SeqSphere^+^ v.8.3.0 software (Ridom GmbH, Münster, Germany) [46]. Genotypes differing in ≤15 alleles were defined as closely related. Isolates with the following STs: ST11, ST14, ST15, ST16, ST17, ST20, ST37, ST48, ST101, ST147, ST258, ST307, ST336, ST340, ST383, ST395, ST512 were classified as HiR clones, i.e., clones that caused at least four recognised outbreaks and were reported in ≥10 countries [1], but also including well described emerging *K. pneumoniae* lineages previously designated as HiR by various researchers [21,23,47,48,49]. 

### 4.5. Antimicrobial Resistance and Virulence Genes

ABRicate v1.0.1 (https://github.com/tseemann/ABRicate (accessed on 1 February 2022)) (with options--minid 90--mincov 90) was used to screen for acquired antimicrobial resistance genes with ResFinder (database date 1 February 2022) [50]. Kaptive, integrated in Kleborate, was used with the default options to assign the *K. pneumoniae* isolates to capsular locus types (KL). In detail, results with a K locus confidence “perfect”, “very high”, “high”, and “good” were counted, while genomes with “low” or “none” confidence Kaptive calls were classified as unknowns. Finally, the same platform was used to screen the isolates for five important acquired virulence loci, i.e., the siderophores yersiniabactin (*ybt*), aerobactin (*iuc*), and salmochelin (*iro*); the genotoxin colibactin (*clb*); and the hypermucoidy locus *rmpADC* [31,51].

## Figures and Tables

**Table 1:** Number of colonising and bloodstream *K. pneumoniae* complex isolates recovered at six study centres in Germany.

Centre	3GCR/CR Colonising Isolates					3GCR/CR Bloodstream Isolates				3GCS/CS Bloodstream Isolates				3GC-Intermediate Bloodstream Isolates
	No. of Isolates by Year of Study					No. of Isolates by Year of Study				No. of Isolates by Year of Study				No. of Isolates
	2016	2017	2018	2019	Total	2016	2017	2018	Total	2016	2017	2018	Total	Total *
A	4 (4)	10 (10)	13 (13)	10 (10)	37 (37)	7 (2)	32 (5)	29 (10)	68 (17)	26 (0)	91 (0)	120 (0)	237 (0)	7 (0)
B	5 (5)	1 (1)	5 (5)	5 (5)	16 (16)	5 (5)	12 (12)	7 (7)	24 (24)	12 (0)	64 (10)	74 (15)	150 (25)	4 (0)
C	6 (6)	4 (4)	5 (5)	4 (4)	19 (19)	0 (0)	13 (12)	7 (7)	20 (19)	12 (0)	57 (12)	62 (20)	131 (32)	6 (0)
D	1 (1)	0 (0)	1 (1)	1 (1)	3 (3)	0 (0)	11 (11)	4 (3)	15 (14)	4 (0)	32 (11)	38 (11)	74 (22)	2 (0)
E	1 (1)	0 (0)	0 (0)	5 (5)	6 (6)	0 (0)	1 (1)	0 (0)	1 (1)	2 (0)	22 (1)	41 (4)	65 (5)	4 (0)
F	nd	4 (4)	5 (4)	2 (2)	11 (10)	0 (0)	2 (2)	3 (3)	5 (5)	4 (0)	27 (5)	33 (6)	64 (11)	2 (0)
Total	17 (17)	19 (19)	29 (28)	27 (27)	92 (91)	12 (7)	71 (43)	50 (30)	133 (80)	60 (0)	293 (39)	368 (56)	721 (95)	26 (0)

Numbers in parentheses represent number of isolates sequenced; nd: no data available as Centre F did not participate in 2016; * 3GC-intermediate bloodstream isolates distribution per year: 2016, *n* = 3; 2017, *n* = 6; 2018, *n* = 17.

**Table 2:** Overview of the most frequent sequence types of colonising and bloodstream *K. pneumoniae* complex isolates excluding most singletons.

Colonising Isolates		Bloodstream Isolates		Bloodstream Isolates	
3GCR/CR (*n* = 91)		3GCR/CR (*n* = 80)		3GCS/CS (*n* = 95)	
ST	No. (%) of Isolates	ST	No. (%) of Isolates	ST	No. (%) of Isolates
ST307 *	10 (11)	ST307 *	13 (16.2)	ST37 *	7 (7.4)
ST45	5 (5.5)	ST15 *	7 (8.8)	ST14 *	3 (3.2)
ST219	5 (5.5)	ST48 *	7 (8.8)	ST17 *	3 (3.2)
ST14 *	4 (4.4)	ST219	5 (6.3)	ST160	2 (2.1)
ST17 *	4 (4.4)	ST147 *	4 (5.0)	ST23	2 (2.1)
ST405	4 (4.4)	ST13	3 (3.8)	ST2599	2 (2.1)
ST15 *	3 (3.3)	ST101 *	3 (3.8)	ST35	2 (2.1)
ST1653	3 (3.3)	ST4	2 (2.5)	ST3640	2 (2.1)
ST20 *	2 (2.2)	ST25	2 (2.5)	ST39	2 (2.1)
ST29	2 (2.2)	ST392	2 (2.5)	ST6069	2 (2.1)
ST37 *	2 (2.2)	ST1825	2 (2.5)	ST641	2 (2.1)
ST48 *	1 (1.1)	ST405	2 (2.5)	ST20 *	1 (1.1)
ST147 *	1 (1.1)	ST607	2 (2.5)	ST101 *	1 (1.1)
		ST17 *	1 (1.3)		
		ST37 *	1 (1.3)		
		ST258 *	1 (1.3)		
		ST383 *	1 (1.3)		
		ST395 *	1 (1.3)		
Other Singletons	45 (49.5)	Other Singletons	21 (26.3)	Singletons	64 (67.4)
Total HiR	27 (29.7)	Total HiR	39 (48.8)	Total HiR	15 (15.8)

* HiR clones.

**Table 3:** Overview of acquired beta-lactamases of colonising and bloodstream *K. pneumoniae* complex isolates.

Acquired Beta-Lactamase		No. of 3GCR/CR Colonising Isolates	No. of 3GCR/CR Bloodstream Isolates	No. of 3GCS/CS Bloodstream Isolates
Extended-spectrum	*bla*_CTX-M-1_	2	1	0
	*bla*_CTX-M-3_	0	1	0
	*bla*_CTX-M-14_	10	2	0
	*bla*_CTX-M-14b_	1	1	0
	*bla*_CTX-M-15_	68	59	0
	*bla*_CTX-M-27_	1	1	0
	*bla*_CTX-M-55_	1	0	0
	*bla*_CTX-M-65_	1	0	0
	*bla*_OXA-1_	24	30	0
Broad-spectrum	*bla*_TEM-1A_	1	3	1
	*bla*_TEM-1B_	46	35	2
Narrow-spectrum	*bla*_OXA-9_	0	4	1
Carbapenemases	*bla*_KPC-2_	0	1	0
	*bla*_OXA-48_	0	2	0
	*bla*_VIM-19_	0	1	0
AmpC	*bla*_CMY-4_	0	1	0
	*bla*_DHA-1_	2	5	0
Total		157	147	4

## Data Availability

The raw sequencing reads generated in this project were submitted to the European Nucleotide Archive (https://www.ebi.ac.uk/ena/) under the study accession number PRJEB39867.

## Supplementary Materials

The following supporting information can be downloaded at https://www.mdpi.com/article/10.3390/antibiotics11101286/s1. Table S1: Overview of the STs of the *K. pneumoniae* complex colonising and bloodstream isolates including singletons. Table S2: Overview of the species, STs, and virulence factors of the *K. pneumoniae* complex 3GCR/CR colonising (a), 3GCR/CR (b), and 3GCS/CS (c) bloodstream isolates. Figure S1. (A) Prevalence of 3GCR *K. pneumoniae* complex carriage of patients on hospital admission per year and centre. (B) Prevalence of 3GCR among 880 *K. pneumoniae* complex bloodstream isolates per year and centre. Figure S2. Dendrogram generated using Ridom SeqSphere+ for the 3GCR/CR colonising *K. pneumoniae* complex isolates including HiR clones (*n* = 91) coloured by study centre and identified by ST type, ignoring missing values. Each box represents one isolate from an individual patient according to sequence analysis of 2358 cgMLST target genes. Figure S3. Dendrogram generated using Ridom SeqSphere+ for the 3GCR/CR *K. pneumoniae* complex bloodstream isolates including HiR clones (*n* = 80) coloured by study centre and identified by ST type, ignoring missing values. Each box represents one isolate from an individual patient according to sequence analysis of 2358 cgMLST target genes. Figure S4. Dendrogram generated using Ridom SeqSphere+ for the 3GCS/CS *K. pneumoniae* complex bloodstream isolates including HiR clones (*n* = 95) coloured by study centre and identified by ST type, ignoring missing values. Each box represents one isolate from an individual patient according to sequence analysis of 2358 cgMLST target genes.
