# Supplementary material for: Surveillance and Genomic Analysis of Third-Generation Cephalosporin-Resistant and Carbapenem-Resistant Klebsiella pneumoniae Complex in Germany
Source: Antibiotics (Basel). 2022 Sep 21;11(10):1286. doi: 10.3390/antibiotics11101286 (PMC9598256; doi:10.3390/antibiotics11101286)
Supplement: Supplementary file 1 [file antibiotics-11-01286-s001.zip › supplementary.pdf]

Supplementary Materials

Table S1: Overview of the STs of the *K. pneumoniae* complex colonizing and bloodstream isolates including singletons.

3GCR/CR colonizing isolates (n=91)		3GCR/CR bloodstream isolates (n=80)		3GCS/CS bloodstream isolates (n=95)	
ST	No. of isolates	ST	No. of isolates	ST	No. of isolates
ST307*	10	ST307*	13	ST37*	7
ST219	5	ST15*	7	ST14*	3
ST45	5	ST48*	7	ST17*	3
ST14*	4	ST219	5	ST23	2
ST17*	4	ST147*	4	ST35	2
ST405	4	ST13	3	ST39	2
ST15*	3	ST101*	3	ST160	2
ST1653	3	ST4	2	ST641	2
ST20*	2	ST25	2	ST2599	2
ST29	2	ST392	2	ST3640	2
ST37*	2	ST405	2	ST6069	2
ST13	1	ST607	2	ST2	1
ST25	1	ST1825	2	ST20*	1
ST35	1	ST8	1	ST22	1
ST36	1	ST11*	1	ST29	1
ST48*	1	ST17*	1	ST30	1
ST76	1	ST37*	1	ST36	1
ST133	1	ST39	1	ST45	1
ST147*	1	ST45	1	ST55	1
ST152	1	ST152	1	ST76	1
ST211	1	ST258*	1	ST86	1
ST231	1	ST268	1	ST101*	1
ST242	1	ST273	1	ST111	1
ST309	1	ST280	1	ST145	1
ST323	1	ST310	1	ST188	1
ST327	1	ST353	1	ST230	1
ST432	1	ST383*	1	ST253	1
ST433	1	ST395*	1	ST268	1
ST449	1	ST420	1	ST279	1
ST469	1	ST552	1	ST292	1
ST502	1	ST870	1	ST317	1
ST513	1	ST1106	1	ST347	1
ST551	1	ST1322	1	ST353	1
ST628	1	ST1999	1	ST355	1
ST631	1	ST3251	1	ST363	1
ST716	1	ST4023	1	ST380	1
ST719	1	ST5588	1	ST530	1
ST776	1	ST6075	1	ST587	1
ST791	1	ST6078	1	ST639	1

ST849	1		ST788	1
ST866	1		ST792	1
ST869	1		ST846	1
ST985	1		ST857	1
ST1040	1		ST882	1
ST1626	1		ST981	1
ST1799	1		ST1107	1
ST1845	1		ST1140	1
ST1962	1		ST1180	1
ST2010	1		ST1540	1
ST2279	1		ST1562	1
ST2407	1		ST1564	1
ST2974	1		ST1608	1
ST3191	1		ST1727	1
ST3249	1		ST1791	1
ST6071	1		ST2355	1
ST6082	1		ST2386	1
ST6083	1		ST2388	1
ST6084	1		ST2843	1
			ST2994	1
			ST3074	1
			ST3379	1
			ST4118	1
			ST4156	1
			ST4585	1
			ST4609	1
			ST4860	1
			ST5682	1
			ST6066	1
			ST6067	1
			ST6068	1
			ST6072	1
			ST6073	1
			ST6074	1
			ST6076	1
			ST6077	1
			ST6079	1
			ST6080	1
Total of STs	58	39		77

*HiR clones

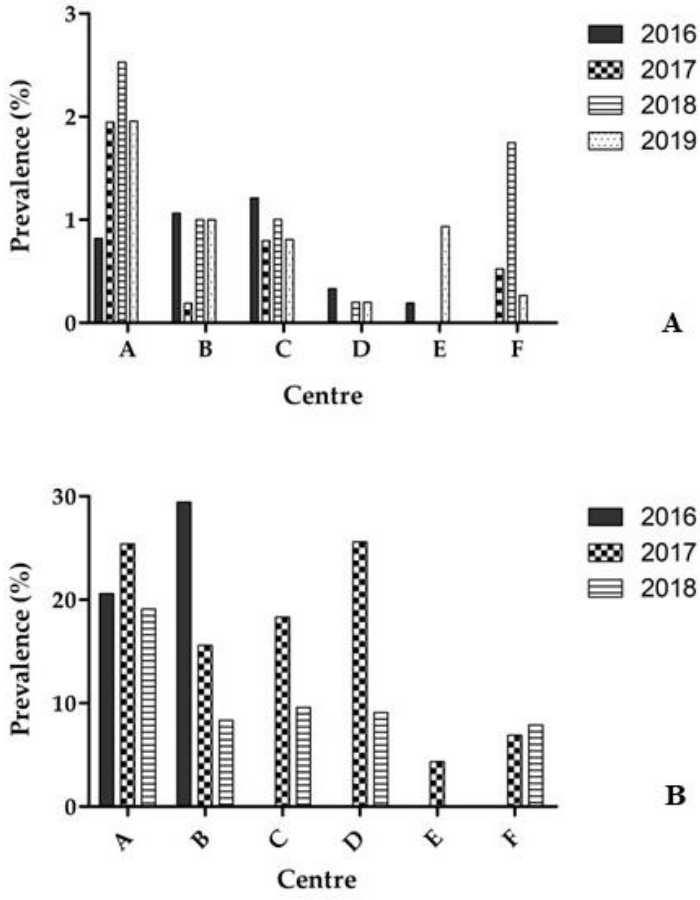

Figure S1. (A). Prevalence of 3GCR *K. pneumoniae* complex carriage of patients on hospital admission per year and centre. (**B**). Prevalence of 3GCR among 880 *K. pneumoniae* complex bloodstream isolates per year and centre.

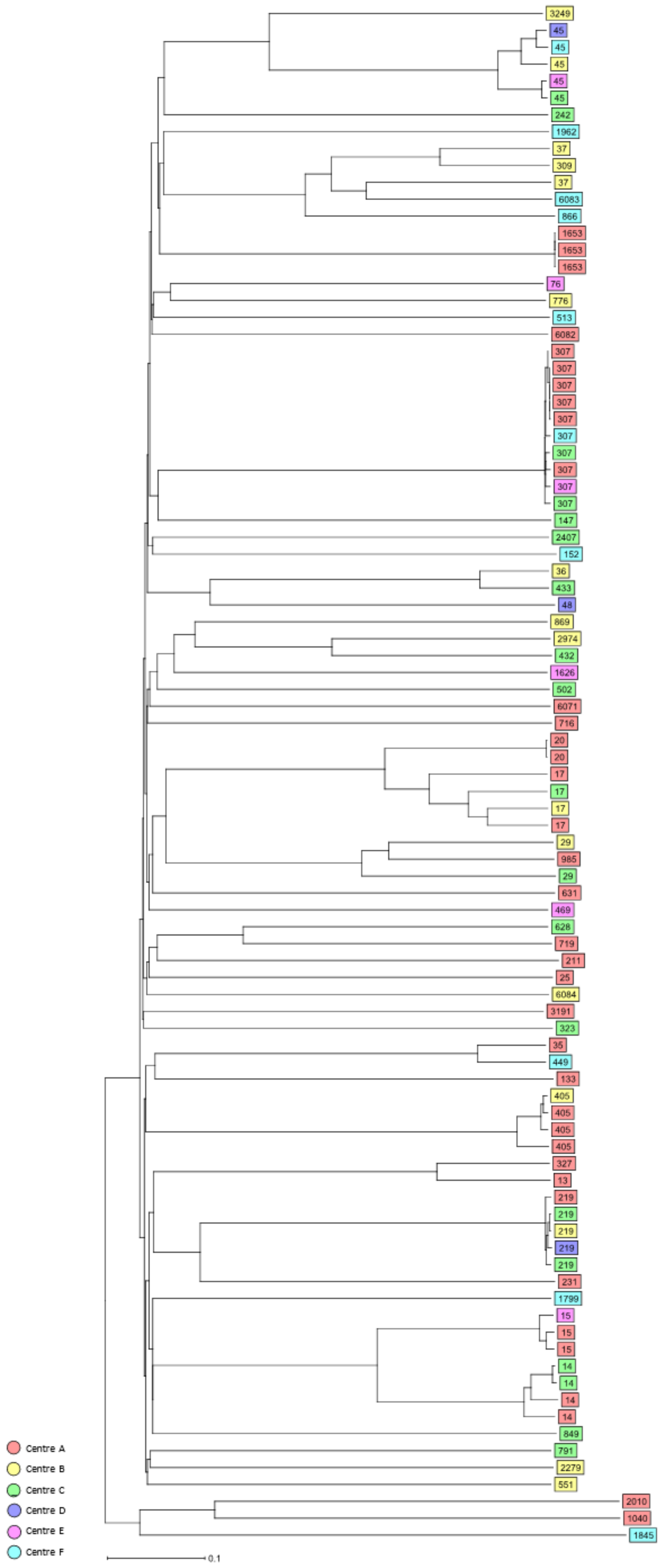

Figure S2. Dendrogram generated using Ridom SeqSphere+ for the 3GCR/CR colonizing *K. pneumoniae* complex isolates including HiR clones (n=91) coloured by study centre and identified by ST type, ignoring missing values. Each box represents one isolate from an individual patient based on sequence analysis of 2358 cgMLST target genes.

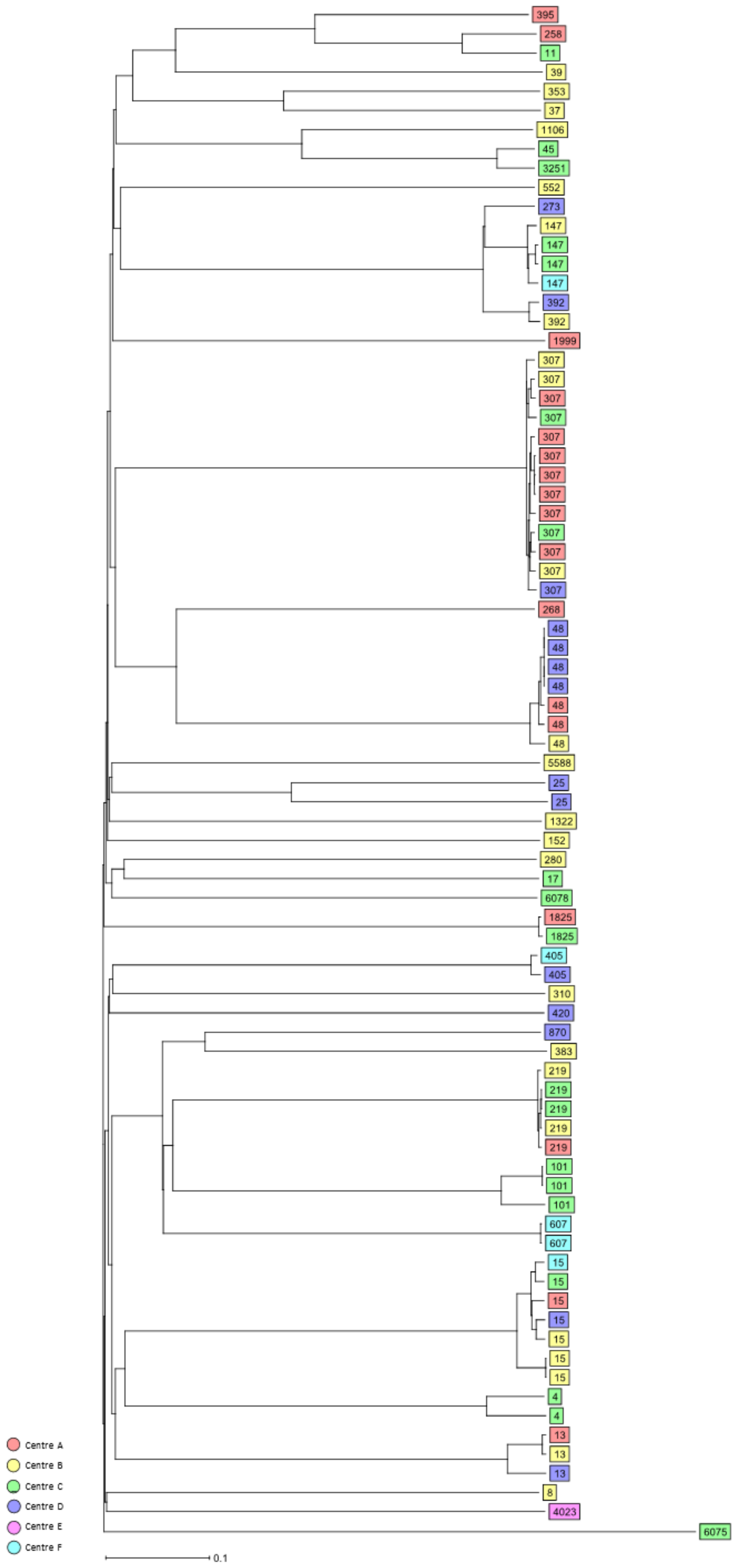

Figure S3. Dendrogram generated using Ridom SeqSphere+ for the 3GCR/CR *K. pneumoniae* complex bloodstream isolates including HiR clones (n=80) coloured by study centre and identified by ST type, ignoring missing values. Each box represents one isolate from an individual patient based on sequence analysis of 2358 cgMLST target genes.

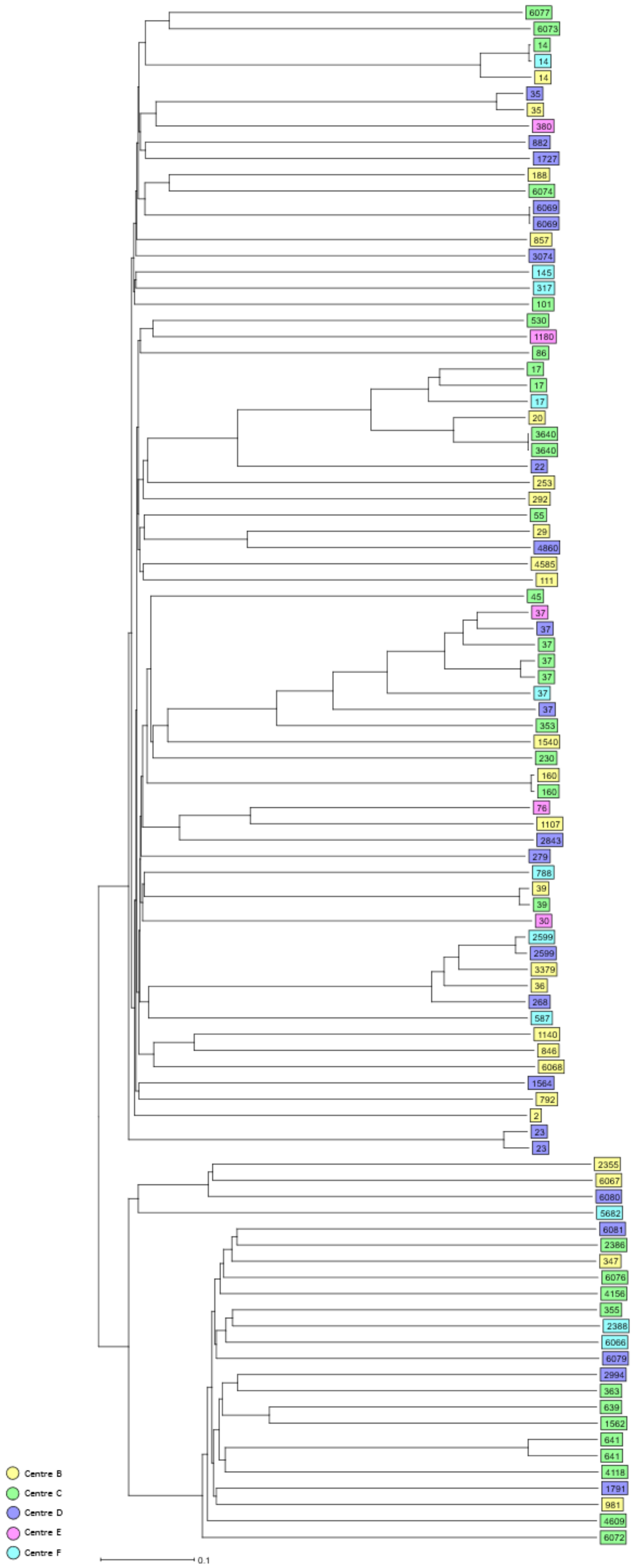

Figure S4. Dendrogram generated using Ridom SeqSphere+ for the 3GCS/CS *K. pneumoniae* complex bloodstream isolates including HiR clones (n=95) coloured by study centre and identified by ST type, ignoring missing values. Each box represents one isolate from an individual patient based on sequence analysis of 2358 cgMLST target genes.
